# A self-learning Monte Carlo tree search algorithm for robot path planning

**DOI:** 10.3389/fnbot.2023.1039644

**Published:** 2023-07-06

**Authors:** Wei Li, Yi Liu, Yan Ma, Kang Xu, Jiang Qiu, Zhongxue Gan

**Affiliations:** ^1^Academy for Engineering and Technology, Fudan University, Shanghai, China; ^2^Ji Hua Laboratory, Department of Engineering Research Center for Intelligent Robotics, Foshan, China

**Keywords:** Monte Carlo tree search (MCTS), path planning, neural network, Markov decision process (MDP), collective intelligent algorithm

## Abstract

This paper proposes a self-learning Monte Carlo tree search algorithm (SL-MCTS), which has the ability to continuously improve its problem-solving ability in single-player scenarios. SL-MCTS combines the MCTS algorithm with a two-branch neural network (PV-Network). The MCTS architecture can balance the search for exploration and exploitation. PV-Network replaces the rollout process of MCTS and predicts the promising search direction and the value of nodes, which increases the MCTS convergence speed and search efficiency. The paper proposes an effective method to assess the trajectory of the current model during the self-learning process by comparing the performance of the current model with that of its best-performing historical model. Additionally, this method can encourage SL-MCTS to generate optimal solutions during the self-learning process. We evaluate the performance of SL-MCTS on the robot path planning scenario. The experimental results show that the performance of SL-MCTS is far superior to the traditional MCTS and single-player MCTS algorithms in terms of path quality and time consumption, especially its time consumption is half less than that of the traditional MCTS algorithms. SL-MCTS also performs comparably to other iterative-based search algorithms designed specifically for path planning tasks.

## 1. Introduction

Path planning is a critical problem in logistics and robotics and has been further applied to many areas (Zhang et al., 2019; Aggarwal and Kumar, 2020; Li et al., 2021). The objective of path planning is to obtain an optimal and collision-free path from the origin to the destination. In recent years, collective intelligence algorithms have been widely used for path planning. These algorithms solve path planning problems by simulating some natural phenomenon or biological behaviors such as particle swarm optimization (Cheng et al., 2021; Halder, 2021; Yu et al., 2022), ant colony optimization (ACO) (Xiong et al., 2021), and genetic algorithm (Lee and Kim, 2016). The collective intelligence algorithm is based on the iterative search to find the solution but typically suffers from poor solution quality, slow convergence and inefficient search (Dai et al., 2019; Cheng et al., 2021).

Monte Carlo tree search (MCTS) is an iterative approach which executes random sampling in the simulation and collects action statistics to enable educated choice in subsequent iterations. Since the number of simulations in each iteration can be considered the number of agents searching in the state space, it is also regarded as a collective intelligence algorithm (Qi et al., 2018, 2021). Agents find a reasonable solution, and then refine it to find an optimal one in the subsequent iteration. One of the most significant advantages of MCTS is that the algorithm does not require domain-specific knowledge, with only search rules specifying which actions are possible and which are terminated in each state. It allows MCTS to be used in any task that can be modeled with decision trees (although it may be helpful to add domain-specific knowledge). Moreover, MCTS can run additional iterations to improve its performance. In particular, MCTS is biased toward more promising states when adding nodes to the search tree. These properties of MCTS make its search process faster than most collective intelligence algorithms. However, with the increasing number of simulations, its search speed also becomes slow. This work proposes an algorithmic framework of self-learning MCTS to address this problem.

MCTS is often adopted in applications, such as games (Crippa et al., 2022), combinatorial optimization problems (Perez et al., 2012), planning problems (Pellier et al., 2010; Dam et al., 2022), and scheduling problems (Huang et al., 2022; Kung et al., 2022). MCTS was initially proposed by Gelly and Wang (2006). Later, Kocsis and Szepesvári (2006) developed MCTS as the first computer Go program, and MCTS rapidly gained widespread attention due to its significant success in playing Go. While some new work applies MCTS and its variations on tasks such as two-player games (Gelly et al., 2012) and multi-player games (Sturtevant, 2008; Scariot et al., 2022), so far there is only a little work about single-player tasks (Schadd et al., 2012). For SameGame, Schadd et al. (2012) proposed Single Player Monte Carlo Tree Search (SP-MCTS) to improve the performance of MCTS on this single-player game. SP-MCTS overperformed previous works in single-player deterministic complete information games by adjusting the selection and back-propagation strategies. Furthermore, Crippa et al. (2022) improved the performance of SP-MCTS in SameGame by solving the deadlock problems. Dam et al. (2022) tried to use MCTS to find feasible solutions in robot path planning. This work shows that a suitable sampling range, hyper-parameter of sampling configuration and exploration strategies could substantially boost the performance of MCTS significantly. In summary, the MCTS algorithms mentioned above are based on the conventional MCTS framework, i.e., they focus on solving a single problem through a large number of random searches in the simulation process, which is a greedy way to find a solution. It leads the search process to be inefficient.

In recent years, the outstanding performance of AlphaGo Zero in playing the game Go (Silver et al., 2016, 2017) further highlighted the capabilities of MCTS. The critical characteristic of AlphaGo Zero is to assess each game's trajectories based on the self-play results. However, self-play in two-player zero-sum scenarios is based on game relationships, and it is not directly transferable to be used in single-player scenarios. The main challenge is evaluating the current model's solution quality in the environment without the game relationship. In this paper, we construct a self-learning approach for single-player tasks, which enables the single-player MCTS to improve its problem-solving ability by learning from its historical experience. The proposed self-learning MCTS (SL-MCTS) combines MCTS with a neural network (PV-Network). The framework of MCTS can balance the exploration and exploitation of search. PV-Network replaces the rollout process of the traditional MCTS framework and predicts the search probability of each subsequent move and the state value, which reduces the operational time of SL-MCTS. This work presents a method to evaluate the performance of the current model's solution for the self-learning process of SL-MCTS by comparing the current model's performance with the solution obtained from the best historical model so far. The current solution is scored higher (lower) if better (worse) than the previous optimal solution. This method can guide PV-Network to make predictions accurately, increasing the effectiveness of SL-MCTS search. SL-MCTS generates training data based on the solutions of the current model and their corresponding scores. In the self-learning process, PV-Network improves its selection probability and score prediction accuracy by learning the historical experience of SL-MCTS. The enhanced PV-Network can, in turn, guide SL-MCTS to find a better solution. The above process is repeated to gradually improve the problem-solving ability of SL-MCTS. In this paper, we validated the effectiveness of the proposed method in the classic and widely used path planning scenario.

The main contributions of this paper are summarized as follows:

1. We propose a self-learning framework to continuously improve the problem-solving ability of SL-MCTS in a single-player environment.
2. This study proposes a method to evaluate decision quality in single-player scenarios, which utilizes the best historical models. By utilizing this evaluation method, the SL-MCTS algorithm can consistently and effectively enhance its decision-making capacity in single-player scenarios.
3. We demonstrate that SL-MCTS effectively improves problem-solving ability through self-learning process in robot path planning scenario. Comparisons with other MCTS algorithms and collective intelligence algorithms also confirm the superior efficiency of SL-MCTS.

The rest of this paper is organized as the following. Section 2 presents the construction of environmental maps and the definition of the path planning problem in this paper, the procedure of conventional MCTS algorithms, and the detail of SL-MCTS algorithm. Section 3 provides the experimental setting and experimental results of SL-MCTS. We also compare the performance of SL-MCTS with traditional MCTS SP-MCTS algorithms and other collective intelligence algorithms in robot path planning scenarios. The paper is concluded in Section 4, where we also discuss ideas for future works.

## 2. Materials and methods

### 2.1. Problem formulation

#### 2.1.1. Path planning problem

**Figure 1:**
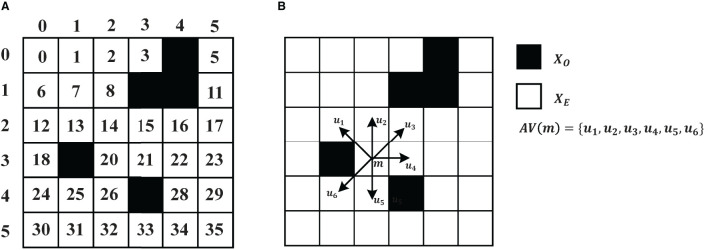
**(A)** Environment model. **(B)** An example of a two-dimensional path planning problem with eight directions.

This paper utilizes the grid model to form the robot's working environment for path planning tasks. As shown in Figure 1A, the space is partitioned into *N*×*N* blocks, whereby the black grids represent obstacles (grids with barriers), and the white grids represent free space (areas where the robot can move). To identify obstacles, white grid cells are represented by 0, whereas back grid units are represented by 1.

Figure 1B is an example of a 6 × 6 grid map. The task information includes a pair of origins and destinations. The set of all nodes is denoted as *X*, where *X*_*O*_ denotes the set of obstacles and *X*_*E*_ contains all the feasible. The origin and destination are respectively denoted as *m*_*s*_ and *m*_*d*_. The relationship between all feasible nodes (*X*_*E*_) is denoted as *G* = (*M, E*), where *M* ∈ *X*_*E*_ and *E* is the edges to neighbor nodes of *M*. *AE*(*m*) = {*m*′∣(*m, m*′) ∈ *E, m*≠*m*′} represents all feasible neighbors of node *m*. *N*(*AE*(*m*)) is the number of the feasible nodes of *m*. The cost of each edge is recorded as 1. Therefore the path planning can be described as an agent starting from position *x*_*s*_ at time step *t*_0_ to position *x*_*d*_. At time step *t*_1_, the agent selects action *a*_1_ and moves to the next state *s*_1_. After *T* steps, the agent reaches the position *x*_*d*_ in *S*_*T*_. The sequential solution is *path* = ((*a*_0_, *a*_1_, …, *a*_*t*_, …, *a*_*T*_), *a*_0_ = *x*_*s*_, *a*_*T*_ = *x*_*d*_, *a*_*t*_ ∈ *AE*(*a*_*t*−1_)), and the path length is $\overset{t=T-1}{\underset{t=0}{\sum }}cost\left(a_{t},a_{t+1}\right)$.

#### 2.1.2. Markov decision process of path planning

**Figure 2:**
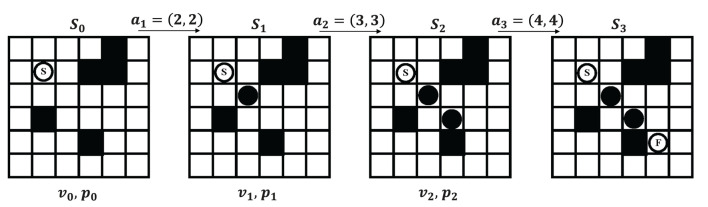
Modeling path planning problem as a Markov decision process.

We model the search process of path planning as a Markov Decision Process (MDP). The process can be described as shown in Figure 2. At each time step, the map is defined as state *S*_*t*_(*t* = 0, 1, 2, 3, ..., *T*). The neural network predicts the state value *v*_*t*_ and the selection probabilities *p*_*t*_ for each state *S*_*t*_. The choice of action *a*_*t* + 1_ is together determined by *v*_*t*_ and *p*_*t*_, executing action *a*_*t* + 1_ and transferring to the next state *S*_*t* + 1_. This process continues until the agent reaches the end.

### 2.2. Monte Carlo tree search

To explicitly compare the differences between the framework of traditional MCTS and that of SL-MCTS, we describe the flow of the traditional MCTS algorithm in this part. For the family of traditional MCTS algorithms, their steps are similar.

**Figure 3:**
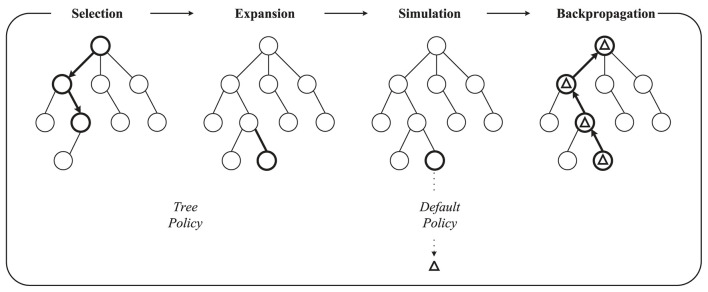
The traditional MCTS in one iteration. This process starts from a root node. Tree Policy is used to select feasible nodes. Default Policy or Rollout Policy is used to rapidly find the result in simulation. Finally, the result Δ is backpropagated to all nodes visited during this iteration.

Two fundamental concepts guide the search process of MCTS algorithms: (1) the true value of an action can be approached by a large number of stochastic simulations; (2) these values can be effectively used to adjust the policy to the best-preferred strategy. MCTS builds a search tree to estimate the values of the moves. These estimates (especially those of the most promising directions) become more and more accurate as the iterative search increases. Generally, the basic MCTS algorithm has four main processes (as shown in Figure 3): selection, expansion, simulation, and backpropagation. The tree policy is used to balance exploration and exploitation in the search and also determines the search direction. The Default Policy aims to calculate the action value of the non-terminal state by rapidly exploring a certain depth of the tree in the rollout. The rollout subtree provides the statistics for MCTS decision-making. The general approach of the rollout is to select actions based on uniform distribution. In the rollout process, a quick search is performed according to the default policy to produce a rollout subtree and find a result until the limits on the maximum number of iterations and the maximum depth of exploration are reached. In general, with a larger number of search depths and iterations, MCTS performs well, but it also causes the problem of inefficient search.

### 2.3. SL-MCTS algorithm

**Figure 4:**
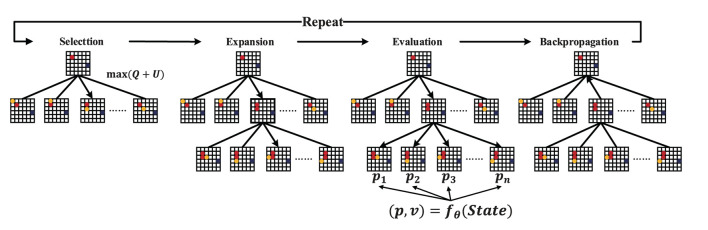
The pipeline of SL-MCTS. *f*_θ_ is PV-Network. The state is the input of the neural network. The output is the selection probabilities *p* of each child node and the state value *v*. The deep blue node indicates the endpoint, the red node indicates the historical route during the search, and the yellow node indicates the feasible space under the current state.

Algorithm 1 presents the pseudocode of SL-MCTS. SL-MCTS combines MCTS with a two-branch neural network (PV-Network) which guides the evaluation phase of SL-MCTS (Figure 4). The search process of SL-MCTS is shown in Algorithm 1, lines 3-12. PV-Network has two branches that output the selection probabilities *p* of all feasible nodes and a state value *v*, respectively (line 7). The selection probability *p* of each node is output after the search (line 10). At the end of the task, the solution score *z* is evaluated by comparing it with the optimal historical model (line 13), which means the quality of paths. The training process is shown in lines 15–20. In the training process, the parameters of PV-Network are updated, which makes the select probability *p* and state value *v* closer to the search probability π and path quality score *z* of previous SL-MCTS (line 16). Finally, these new network parameters are used in the next iteration of self-learning to make the search direction of SL-MCTS more accurate. The map and historical path information are fused as input state *S*_*t*_. The selection probability *p* is a vector. It enables the quick search process of SL-MCTS to be more efficient than MCTS. The state value *v* is a scalar representing the path quality in each direction predicted at this position. It guides SL-MCTS toward the best-preferred strategy.

**Algorithm 1:**
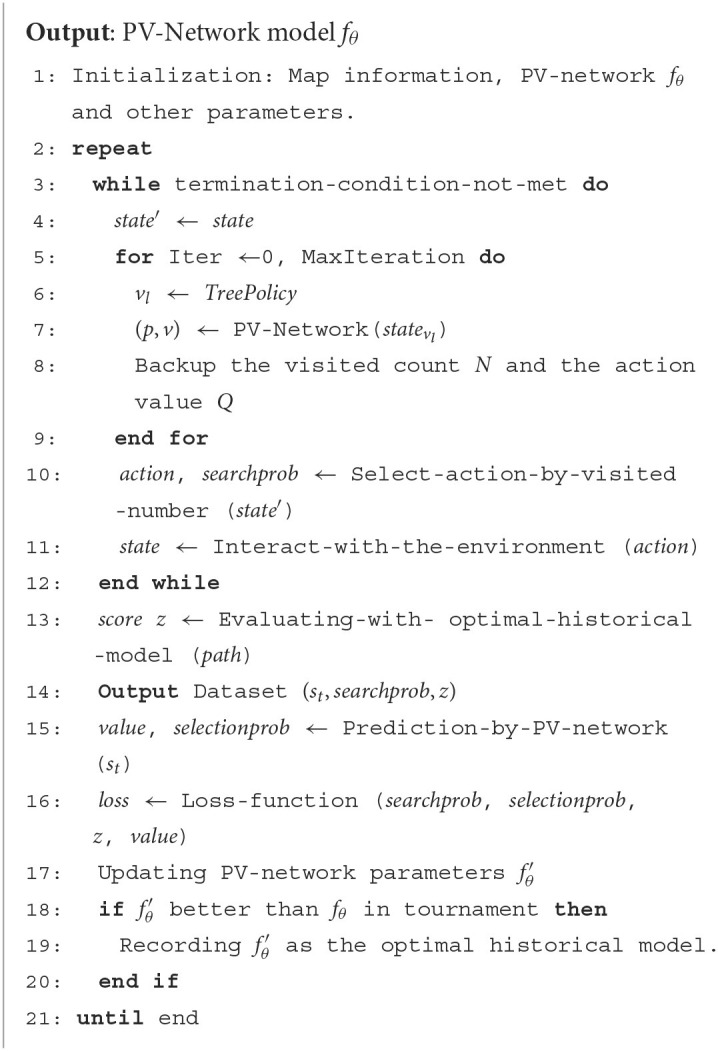
SL-MCTS path planning algorithm.

The pipeline of SL-MCTS is shown in Figure 4. It includes four steps: Selection, Expansion, Evaluation and Backpropagation. Suppose that at time step *t*, the agent is at node *m*_*t*_. Regard *m*_*t*_ as the root node. One iteration of SL-MCTS at time step *t* is as follows:

1. Selection. If *m*_*t*_ is not a leaf, the agent uses Tree Policy to descend through the search tree until the most urgent expandable node is found. The Tree Policy of SL-MCTS is represented by Eqs (1) and (2). Equation (1) balances between exploitation ($\bar{Q}\left(x\right)$) and exploration (*U*(*s*_*t*_, *x*)) of search.

(1) $$m_{next}=\underset{x\in AE(m_{t})}{\text{arg max}}(\bar{Q}(x)+U(s_{t},x))$$

(2) $$\begin{array}{l} U\left(s_{t},x\right)=\frac{C_{puct}P\left(s_{t},x\right)\sqrt{\ln N\left(m_{t}\right)}}{1+N\left(x\right)} \end{array}$$

where *m*_*t*_ is the location of agent in the search tree; *s*_*t*_ is the environment information at *m*_*t*_; *x* is the child of *m*_*t*_, *x* ∈ *AE*(*m*_*t*_); *P*(*s*_*t*_, *x*) is the selection probability of each child node *x* and is one of the predictions of PV-Network; *AE*(*m*_*t*_) is a set of legal action for *m*_*t*_; *N*(*m*_*t*_) is the visit count of *m*_*t*_; *N*(*x*) is the visit count of *x*; *C*_*puct*_>0 is a hyperparameter, which means the amount of exploration performed; *m*_*next*_ is the branch selected for further exploration.

2. Expansion. If *m*_*t*_ is a leaf node, the available neighbor node(s) *AE*(*m*_*t*_) are added to expand the search tree.
3. Evaluation. PV-Network predicts the state value *v* and the selection probability *p* in the iteration.
4. Backpropagation. The visited count and action value *Q* are backpropagated through the search tree to update nodes' statistics. The *Q* value corresponds to the aggregate reward of all rollouts that pass through this state. The statistics are update by Eqs 3) and (4):

(3) $$\begin{array}{l} N{\left(m_{n}\right)}^{\prime }=N\left(m_{n}\right)+1 \end{array}$$

and


(4) $$\begin{array}{l} {\bar{Q}}^{\prime }=\frac{N\left(m_{n}\right)\times \bar{Q}+v}{N{\left(m_{n}\right)}^{\prime }} \end{array}$$

where *m*_*n*_ is one node in the search tree. $\bar{Q}$ is the action value of *m*_*n*_ before it is updated; ${\bar{Q}}^{\prime }$ is the value after it updates; *N*(*m*_*n*_) is the visited number of *m*_*n*_; The state value *v* is one of the outputs of PV-network.

When the iteration limit has been reached, the next move *m*_*t* + 1_ is selected from node *m*_*t*_ based on the search probability π of SL-MCTS:


(5) $$\pi (a|s_{t})=\underset{a}{\text{arg max}}\frac{N(a)}{N(m_{t})},a\in AE(m_{t})$$

where *a* is the child node of *m*_*t*_; *N*(*a*) is the visited count of node *a*.

SL-MCTS differs from the Simulation phase of the traditional MCTS algorithm. PV-Network replaces the rollout process in the traditional MCTS algorithm and can predict the selection probability of the feasible nodes and the state value. SL-MCTS has a more efficient search process and a more accurate search direction.

#### 2.3.1. PV-network

**Figure 5:**
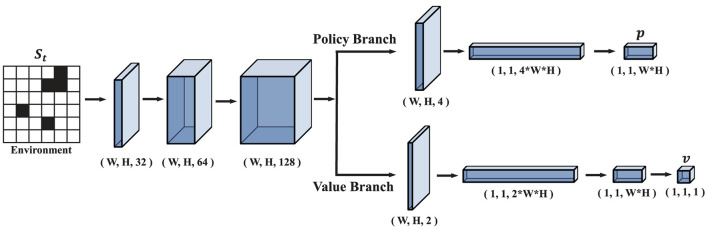
The architecture of PV-Network. *W* is the width of the map and *H* is the height of the map. *p* is the output of the policy branch and *v* is the output of the value branch.

The architecture of PV-Network is shown in Figure 5. PV-network consists of a backbone and then is divided into a policy branch and a value branch to output the selection probability *p* and the state value *v*. The backbone consists of three convolutional layers, and the kernel size is 3 × 3 with stride one and activated by the ReLU function. This network utilizes the convolutional layers to extract local information on the map, followed by fully connected layers to extract global information. The number of channels of these three convolutional layers in the backbone is 32, 64, and 128, respectively. The output of the backbone is used as input to the policy branch and value branch. The policy branch outputs a vector *p*. The value branch outputs a scalar, *v*.

**Figure 6:**
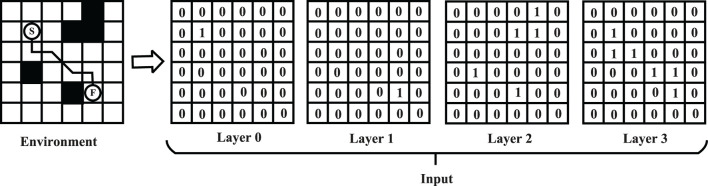
Transformation process from map information to input features.

Figure 6 represents transforming from map information to the input features of PV-Network. The size of input *S*_*t*_ is *n*×*n*×4 where *n*×*n* is the map size. The input comprises four binary feature matrices. The first matrix represents the start position of the task (Figure 6, Layer 0); the second represents the end position (Layer 1); the third represents the position of all obstacles on the map (Layer 2); the fourth represents the position of the nodes on the historical route (Layer 3). The four metrics are represented by “1” for existence and “0” for non-existence. For example, in Layer 3 in Figure 6, the node on the path is noted as “1” and the other as “0.” *p* is a vector including the probability of the feasible nodes at *S*_*t*_. The state value is a scalar in the range of (0, 1).

#### 2.3.2. The framework of self-learning

Self-learning is the process of SL-MCTS generating data for training and gradually improving its decision-making ability by learning those data. Firstly, the initial model is recorded as the optimal historical model. Then, the quality of SL-MCTS's solutions is evaluated using the optimal historical model. A higher score is given to the solution of the current model if it is better than the existing model. As a result, the current model is recorded as the optimal historical model, and the optimal historical mode is generally updated during the training. The data for model training is generated based on the solutions and scores. Repeating the above process, SL-MCTS improves its ability to find the optimal path and generates better training data.

**Figure 7:**
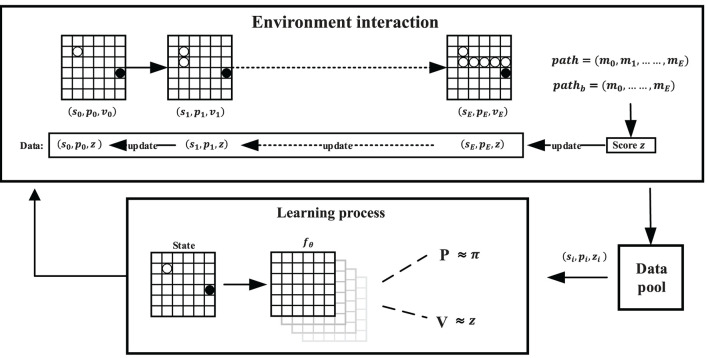
Training pipeline of SL-MCTS algorithm. path is the result of SL-MCTS algorithm in the training process. *path*_*b*_ is the path planning result of SL-MCTS with the optimal historical model, which is used to assess the path score of *path*. The outcome of the assessment is recorded as path score *z*. The historical experiments are saved in the training data set. The beginning and destination positions of *path* and *path*_*b*_ are the same, but the path lengths may differ. *m*_0_ and *m*_*E*_ are the beginning and destination of the example task in this figure. SL-MCTS generates training data by solving many tasks with different beginning and destination positions.

The detail of the self-learning framework is shown in Figure 7. The beginning and destination of the task represent *m*_0_ and *m*_*E*_, and the parameters of the PV-Network *f*_θ_ are denoted by θ. The initialization state of each task is noted as *s*_0_. The Evaluation process of SL-MCTS makes sampling based on the predicted selection probabilities *p* and the state value *v* by the network *f*_θ_. Then, SL-MCTS selects a node *m*_1_ to move and transfer from *s*_0_ to *s*_1_. The search finishes until the endpoint *x*_*d*_ is reached. As shown in Figure 7, SL-MCTS generates a path *path*. The quality of its path is evaluated by the result of the optimal historical model to get a score *z*. The optimal historical model is the best model based on the evaluation method of the Elo rating system (details in Section 3.2) during the training process. SL-MCTS with the optimal historical model produces a result of *path*_*b*_. The path score is calculated depending on Eqs (7) and 8). *path* is split into data of the format (*s*_*t*_, *p*_*t*_, *z*) based on the number of nodes. These data are independent and are stored in the training data set. In the training process, SL-MCTS solves many random tasks and generates data. Lastly, PV-network is trained by randomly sampling the training data set in a small batch. This method of splitting data can significantly break the association between paths and improve the algorithm's stability.

The loss function of PV-Network is:


(6) $$loss={\left(z-v\right)}^{2}-\;\pi^{T}\log\;p+c{\left\|\theta \right\|}^{2}$$

where *c* is a hyperparameter controlling the level of L2 weight regularization, which is to prevent overfitting and controls the contribution of the regularization term to the loss function. The network parameters θ are adjusted based on the loss function Eq. (6) to minimize the error between the predicted state value *v* and path score *z* and to maximize the similarity between the selection probability *p* and the search probability π.

To expand the range of exploration of SL-MCTS in the training process and avoid falling into the local optimal trap, Dirichlet noise is added to the selection probability *p*(*s, a*) ← (1−ε_1_)*p*(*s, a*) + ε_1_η_*a*_, where *s* is the state, *a* is legal action, and *p*(*s, a*) is the predicted selection probability of each *a*. ε_1_ is set to 0.5, and it is used to encourage the exploration of different actions. Dirichlet noise is also added into the search probability π ← (1−ε_2_)π + ε_2_η_*a*_, where η_*a*_ ~ *Dir*(0.3) and ε_2_ is 0.25, to encourage SL-MCTS to explore every feasible node during the training process. The higher ε_2_ is, the more different states are explored and thus enhance the data diversity of the PV-Network.

In the path planning task, the path evaluation is not only related to whether the endpoint is reached but also considers the length of the path. Using only Euclidean distance or Manhattan distance is not reasonable to evaluate path quality. This method can not reflect the existence of obstacles on the line between two points and provides the agent with ambiguous feedback that does not reflect changes in the quality of its solution. Therefore, SL-MCTS generates a path score representing the current problem-solving ability by comparing their results with the optimal historical model. The path score is given by Eqs (7) and (8):


(7) $$\begin{array}{l} l=len\left(path_{b}\right)-len\left(path\right) \end{array}$$

(8) $$\begin{array}{l} z=\frac{2}{1+e^{-\gamma l}}-1 \end{array}$$

where γ ∈ (0, 1]. If the result of Eq. (7) is < 0, it denotes that the solution of the optimal historical model is better than the solution of the current model. *path* receives a score under zero, which means that similar decisions are discouraged. In contrast, if the result of Eq. (7) exceeds 0, indicating that the path length of the optimal historical model is longer than that of the current model, *path* receives a score above zero, which means that those similar decisions are encouraged. Furthermore, if SL-MCTS with the current model fails to reach the destination, this *path* receives a score, −1. The evaluator of SL-MCTS is dynamically adjusted according to the update of the optimal historical model during the training process.

### 2.4. Computational complexity

As there are many different tasks in path planning, it is difficult to assess the computational complexity accurately. The computational complexity of SL-MCTS is analyzed by referring to the calculation method in Yonetani et al. (2021) and Qi et al. (2021). The difference in computational complexity between SL-MCTS and MCTS is mainly in the simulation phase at each time step. Therefore, the analysis focuses on the differences in their computational complexity during the simulation phase. Suppose the length of the path is *l*, *a* is the feasible space for each node, and *k* is the number of simulations per search process. For the traditional MCTS algorithm, the maximum search depth in the rollout process is *d*, and its computational complexity is denoted as O(lk(ad)). The computational complexity of PV-Network is defined as O(|V|) in the training process, according to Yonetani et al. (2021). After training, the computational complexity of the SL-MCTS inference phase is O(lka) and O(lk) for worst and best cases.

## 3. Experiments and analysis

This section provides detailed descriptions on the experimental settings, parameter adjustments, and evaluation methods. We conducted the training process of SL-MCTS on maps with different scales and analyzed the variability of its problem-solving capability. Additionally, we compared the performance of SL-MCTS with other advanced single-player MCTS algorithms and collective intelligence algorithms. Furthermore, we verified the generalization of SL-MCTS on random layout maps with specific obstacle densities and the dynamic environmental map. Finally, we conducted ablation experiments to explore the impact of different simulation times on SL-MCTS. The open-source code, experimental data, and detailed visualizations of the experimental data and results can be found in Liu (2023).

### 3.1. Experimental settings

These experiments were implemented in Python 3.7 using PyTorch. They were executed on a high-performance computing server, using two GeForce RTX 2080 SUPER GPUs for algorithm training in parallel and CPUs that are 3.20 GHz with 16GB memory. The number of simulations of SL-MCTS is set to 30 and *C*_*puct*_ is $1/\sqrt{2}$. The Adam optimizer optimizes the neural network. The learning rate is 10^−3^, and its initial multiplier (*lr*_*m*_) is 1.0. To avoid updating the policy parameters too much at each training iteration, the KL divergence (Nielsen, 2020) is used to adjust *lr*_*m*_ to improve the training stability. Referring to the Proximal Policy Optimization algorithm (Schulman et al., 2017), the probability distributions generated before and after policy updating (*p*_*old*_ and *p*_*new*_) are used to calculate their KL divergence based on the result of Eq. (9). *lr*_*m*_ is adjusted by Eq. (10).


(9) $$\begin{array}{l} KL\left(p_{old}∥p_{new}\right)=\sum p_{old}\cdot log\frac{p_{old}}{p_{new}} \end{array}$$

(10) $$lr_{m}=\{{}_{\frac{lr_{m}}{1.5},\;if\;KL\;>\;2\text{​}\cdot kl_{targ}\;and\;lr_{m}\;>\;0.1}^{1.5\text{​}\cdot \text{​}lr_{m},\;if\;KL\;<\;\frac{kl_{targ}}{2}\;and\;lr_{m}\;<\;10}$$

where the parameter *kl*_*targ*_ is 0.02.

In order to investigate the performance of SL-MCTS on environmental maps of varying scales, we conducted experiments on 6 × 6 and 16 × 16 maps, respectively. The size of the training data set is 10,000. If the data set is completely full, older data is automatically removed as newer data are added Positive samples are defined as those paths that reach the destination and achieve equal to or shorter lengths than the optimal historical model's results. To provide a high-quality training data set for the initial training process of SL-MCTS and rapidly promote the ability of SL-MCTS, the positive sample and negative sample is stored by a 1:1 ratio in the training data set at the initial stage of training.

In this paper, SL-MCTS algorithm compares with variants of MCTS like UCB1 (Auer et al., 2002), MCTS (or UCT) (Kocsis and Szepesvári, 2006) and the variations of SP-MCTS (such as those presented in Schadd et al., 2012; Crippa et al., 2022), to verify its performance. SP-MCTS-CRIPPA (Crippa et al., 2022) is one of the best single-player MCTS algorithms. Additionally, this paper compares SL-MCTS algorithm to prevailing collective intelligence algorithms, including ACO algorithm (Dorigo et al., 2006) and PPACO algorithm (Luo et al., 2020). PPACO is an improved ACO algorithm for path planning problems, which is one of the best ACO algorithms for solving path planning. It has domain-specific knowledge.

### 3.2. Evaluation method

Elo rating system (Coulom, 2008) is used to evaluate the variation of SL-MCTS's problem-solving ability in the training process. The initial Elo ratings of algorithms are 1,000. MCTS-50 and MCTS-150 (Kocsis and Szepesvári, 2006) were chosen for comparison with SL-MCTS, where the number of them denotes the number of simulations. The solution of MCTS-150 is generally better than that of MCTS-50 because the MCTS algorithm can improve its problem-solving capabilities by increasing the number of simulations and the depth of exploration. In this paper, we define the case where SL-MCTS finds the destination, and the path is shorter than the competitor as a win; the case where it finds the destination but the path length is the same as the competitor as the tie; otherwise, it is considered as the failure. The two algorithms update their rating by a “shorter path finding” tournament, which consists of 100 different random tasks. The details of updating the rating are as follows. The expected score of player *a* is presented as


(11) $$\begin{array}{l} E_{a}=\frac{1}{1+10^{\frac{R_{b}-R_{a}}{400}}} \end{array}$$

and the expected score of player *b* is


(12) $$\begin{array}{l} E_{b}=\frac{1}{1+10^{\frac{R_{a}-R_{b}}{400}}}, \end{array}$$

where *R*_*a*_ is the rating of player *a*. After the tournament, if the actual rating of player *a* (*S*_*a*_) differs from its expectation of *E*_*a*_, the level *R*_*a*_ is adjusted as follows:


(13) $$\begin{array}{l} R_{a}^{{}^{\prime }}=R_{a}+K\left(S_{a}-E_{a}\right), \end{array}$$

where *K* is the hyperparameter, which means the range of changes in Elo rating. In this paper, the algorithm's high rating means that it wins more times than its opponent in the tournament, i.e. most of its path lengths are shorter than its opponent's.

To assess the performance of SL-MCTS on the path planning problem, we compared the average path length, time consumption, the standard deviation of path lengths (SD-L) and time consumption (SD-T), visited range and the percentage of successfully solved tasks (Success rate). A smaller average path length reflects a better solution quality of the algorithm. Average time consumption reflects the algorithm's efficiency in solving problems. SD-L and SD-T reflect the variation of the algorithm in the quality and efficiency of solutions. The visited range represents the ratio between the number of visited nodes and the total number of feasible nodes in the map. The success rate is defined as the proportion of successfully completed tasks to the total number of tasks and serves as one of the criteria of the algorithm's problem-solving performance. We also employed the Mann-Whitney U test as a significance test to determine the mean difference between the experimental results for algorithms. The significance level is set to 0.05.

### 3.3. Results and discussion

#### 3.3.1. Performance of self-learning

SL-MCTS's self-learning performances in two scale environmental maps are respectly present. One hundred tasks with different origins and destinations are randomly selected as a tournament from each environment. We used the Elo rating to illustrate the variation of SL-MCTS's problem-solving ability. The initial rating of the Elo rating system (Detailed in Section 3.2) is set to 1,000.

**Figure 8:**
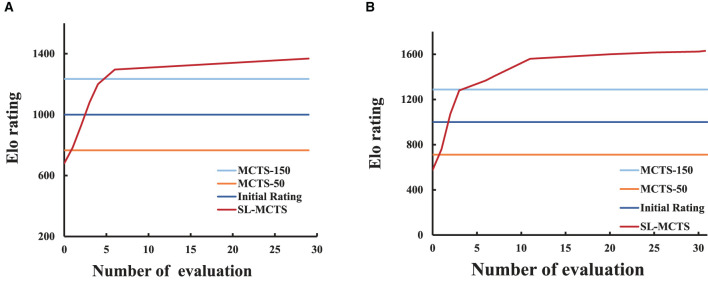
The Elo rating curves of SL-MCTS algorithm. **(A)** Represents the Elo rating curve for SL-MCTS in the 6 × 6 map. **(B)** Represents the Elo rating curve for SL-MCTS in the 16 × 16 map.

Figure 8A shows the Elo rating curves of SL-MCTS, MCTS-50 and MCTS-150 in an obstacle-free 6 × 6 environmental map. Figure 8B shows the performance of SL-MCTS in the 16 × 16 map, which includes 211 feasible nodes and 45 obstacle nodes (as shown in **Figure 10**). As traditional MCTS (Kocsis and Szepesvári, 2006) has no ability to learn the history experiment, its Elo rating is not changed. As shown in Figure 8A, the Elo rating score of MCTS-150 is 1,234, while that of MCTS-50 is 766. In contrast, SL-MCTS algorithm has a considerably lower rating of 680 before any training has taken place, in contrast to the other two traditional MCTS algorithms. At the 1*th* evaluation in the training process of the SL-MCT, the rating of SL-MCTS is 904, which is higher than MCTS-50. At the 7*th* evaluation, its Elo rating is 1,240, which has already exceeded MCTS-50 and MCTS-150. These results indicate that the problem-solving capability of SL-MCTS in the 6 × 6 map is better than MCTS algorithms at 7*th* evaluation. Eventually, the Elo rating of SL-MCTS is 1,368. This value is approximately twice the original Elo rating of the SL-MCTS. As shown in, Figure 8B, the Elo rating of MCTS-50 is 712, and the Elo rating of MCTS-150 is 1,288. The Elo rating of the SL-MCTS algorithm is 576 before the training process, much lower than MCTS. The Elo rating of the SL-MCTS at the 1*th* evaluation exceeds the rating of MCTS-50, which is 760. At the 3*th* evaluation, SL-MCTS's Elo rating is 1,280, which is much similar to that of MCTS-150. The rating of SL-MCTS exceeds that of MCTS-150 at the 6*th* evaluation. The Elo rating of SL-MCTS finally reaches 1,632, which is almost triple the initial rating of SL-MCTS. These results show that the performance of SL-MCTS in the 16 × 16 map is better than MCTS algorithms at 6*th* evaluation. In conclusion, the experimental results in Figure 8 indicate that SL-MCTS performs much worse than MCTS-50 in the beginning (the maximum difference in their Elo rating is 136), which indicates SL-MCTS's initialized PV-Network cannot compete with the rollout process of conventional MCTS. Through self-learning, the Elo rating of SL-MCTS exceeds that of MCTS-50 at the first evaluation in both size environmental maps and exceeds that of MCTS-150 at about the seventh evaluation. Finally, after several training iterations, the Elo rating of SL-MCTS increased approximately three-fold from its initial Elo rating. This also implies that the performance of SL-MCTS significantly enhanced via the self-learning process. The experimental results suggest that SL-MCTS, guided by the PV-Network, can navigate toward a more efficient direction in comparison to the traditional MCTS's rollout process, ultimately leading to better solutions.

**Figure 9:**
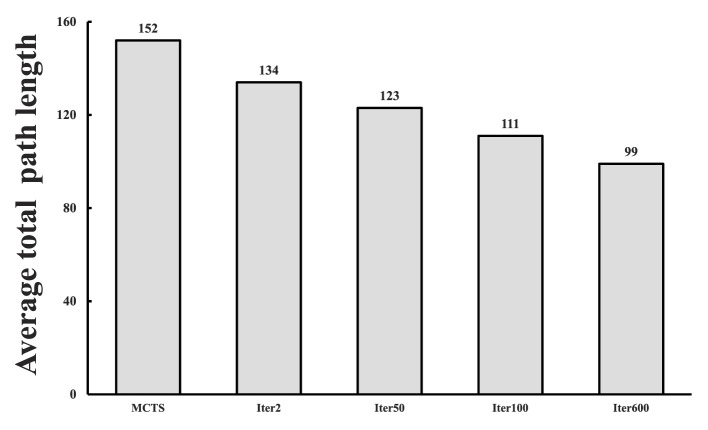
The average total path length of SL-MCTS in different training stages (as shown in columns 2, 3, 4, 5) compared with that of MCTS-50 (As shown in column 1).

To further verify the variation of SL-MCTS's path-finding capability in the self-learning process, we randomly selected 50 tasks in the 6 × 6 map as a test set and compared the average total path length of SL-MCTS at different training stages with that of MCTS-50 (as shown in Figure 9). For each algorithm, the experiments were conducted five times on the test set, using the same parameters. The average of these experiments was used to determine the average total path length (*path*_*at*_) of algorithm, which is calculated by:


(14) $$\begin{array}{l} path_{at}=\frac{1}{5}\overset{5}{\underset{i=1}{\sum }}\overset{50}{\underset{j=1}{\sum }}len^{ij}, \end{array}$$

where *i* represents the times of repeated experiments, while *j* denotes the number of tasks within the test set. *path*_*at*_ for MCTS-50 is 152. Figure 9 illustrates the *path*_*at*_ values generated by SL-MCTS at various learning stages. During the second training iteration, SL-MCTS generated an *path*_*at*_ value of 134, which is comparatively shorter than that of MCTS-50. The *path*_*at*_ value of SL-MCTS shows a decreasing trend as the number of training iterations increase. In particular, the final *path*_*at*_ of SL-MCTS compared to that in the second training iteration decreased by 26%. The evidence of Figures 8, 9 implies that SL-MCTS has significantly improved its path-finding capacity through the process of self-learning.

**Figure 10:**
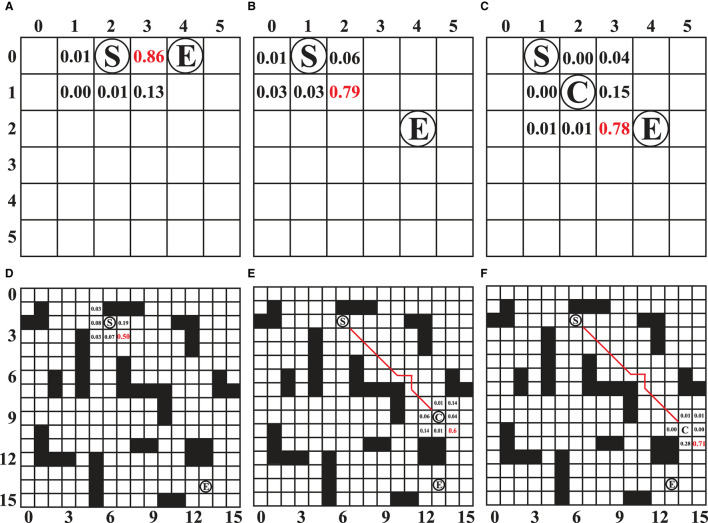
**(A–F)** The predicted selection probability of SL-MCTS for different states. “S” represents the start and “E” represents the destination of the task. “C” represents the position of the agent in the state. Where the value of the number represents the predicted selection probability value of SL-MCTS. The number in red indicates the position with the highest value in the predicted result.

In order to further investigate the guiding role of the PV-network during the reasoning process of SL-MCTS, the predicted probability results of feasible action selection in each step of SL-MCTS were analyzed in this section. As shown in Figure 10, the number on the map means the predicted probability and guides the search direction. “S” represents the start and “E” represents the destination of the task. “C” represents the position of the agent in that state. Figures 10A–C present the three states of the two tasks in 6 × 6 map. Figures 10D–F present the three states of one task in 16 × 16 map. Figure 10A shows that the probability of nodes close to the side of node E is significantly higher than nodes far from node E. The selection probability of node (3, 0) is 0.86, the highest value at that state. Figures 10B, C are the two states of another task which starts at (0,1) and ends at (2,4). The agent starts from node S in Figure 10B, and the agent is at node C in Figure 10C.

In Figure 10B, the maximum probability value is 0.79 at the node (1, 2). The agent executed action (1, 2) and transferred to the next state, as shown in Figure 10C. The node (2, 3) has the highest probability of 0.78 in this stage. The prediction results of SL-MCTS (shown in Figures 10A–C) all present that the nearest node to the destination has the highest selection probability. Figures 10D–F show the three states of one task in the 16 × 16 map, which starts at node (2, 6) and ends at node (14, 14). In Figure 10D, the agent starts from node S. Node (3, 7) has the highest selection probability of 0.5. The agent in Figure 10E is at point C, and the selection probability of (10, 14), which is closest to (14, 14), is the highest, and others are low; Figure 10F is the next state of Figure 10E, where the selection probability of node (11, 15) is the highest. The results in Figure 10 show that the well-trained PV-Network can provide a reasonable selection probability for SL-MCTS based on the global information of the map environment and the current location.

#### 3.3.2. Comparative experiments

SL-MCTS is compared with UCB1 (Auer et al., 2002), MCTS (Kocsis and Szepesvári, 2006), SP-MCTS (Schadd et al., 2012), and SP-MCTS-CRIPPA (Crippa et al., 2022) to show its performance in path planning. The comparison algorithms include UCB1-50, UCB1-150, MCTS-50, MCTS-150, SP-MCTS-50, SP-MCTS-150, SP-MCTS-CRIPPA-50, SP-MCTS-CRIPPA-150 where the numbers indicated the number of simulations. SL-MCTS is also compared with the prevailing collective intelligence algorithm, ACO (Dorigo et al., 2006) and PPACO (Luo et al., 2020). The comparison algorithms included ACO-15-15, and ACO-30-30, where the numbers indicate the number of populations and iterations of ACO. The parameters of the ACO algorithms are set as follows: α = 1, ρ = 0.3, β = 1. We chose three tasks with different origins and destinations: (1, 0) to (8, 0), (2, 14) to (7, 3), and (14, 2) to (6, 15). The span of the tasks' beginning and destination is increasing, which means that the task's difficulty is increasing. This is because, for the algorithm, a larger task span means that it needs to explore a wider area and potentially deal with more obstacles, making it more challenging to search for the destination. The algorithms' shortest path length (Best) in the fifty times of repeated testing, results of the average path length, the average time consumption, the visited range, the standard deviation of path length (SD-L) and time consumption (SD-T), the success rate of finding the destination and p-value after executing the task 50 times are shown in Table 1.

**Table 1:** Performance of UCB1, MCTS, SP-MCTS, SP-MCTS-CRIPPA, ACO, PPACO, and SL-MCTS algorithms on different tasks.

**Algorithm**	**Best**	**Average length**	**Average time(s)**	**Visited range(*%*)**	**SD-L**	**SD-T**	**Success ratio(*%*)**	***p*-value**
**Task 1**								
UCB1-50	**10**	16.42	6.93	100	3.52	1.60	1	1.44 × 10^−17^
UCB1-150	**10**	14.1	19.00	100	2.22	3.50	1	1.03 × 10^−5^
MCTS-50	12	16.92	4.16	100	3.43	0.90	1	2.26 × 10^−3^
MCTS-150	**10**	14.41	18.33	100	2.59	3.59	0.99	2.12 × 10^−3^
SP-MCTS-50	12	19.08	8.35	100	8.34	5.09	1	1.12 × 10^−11^
SP-MCTS-150	12	18.84	24.41	100	4.00	6.63	1	5.93 × 10^−24^
SP-MCTS-CRIPPA-50	11	15.3	6.54	100	3.22	1.45	0.92	8.33 × 10^−15^
SP-MCTS-CRIPPA-150	12	15.5	21.48	100	2.17	3.49	0.94	2.73 × 10^−24^
ACO-15-15	11	99.52	11.00	6.24	**0.52**	0.68	1	3.99 × 10^−5^
ACO-30-30	**10**	***10.68***	10.05	98.10	0.69	0.94	1	–
PPACO-30-30	11	11.58	6.24	99.22	1.04	0.68	1	0.007
SL-MCTS-30	**10**	**11.08**	**2.99**	**14.69**	0.59	**0.04**	1	**1.85 × 10^−1^**
**Task 2**								
UCB1-50	17	28.2	13.04	100	5.96	2.92	1	1.60 × 10_−26_
UCB1-150	17	24.78	41.16	100	5.26	10.96	1	3.13 × 10_−21_
MCTS-50	16	39.08	17.59	100	11.04	6.67	1	2.92 × 10^−27^
MCTS-150	16	38.94	64.97	100	9.13	20.66	1	8.04 × 10^−33^
SP-MCTS-50	**12**	19.08	8.35	100	8.34	5.09	1	6.15 × 10^−4^
SP-MCTS-150	**12**	18.84	24.41	100	4.00	6.63	1	7.03 × 10^−10^
SP-MCTS-CRIPPA-50	30	42.36	20.75	100	9.18	4.91	0.76	1.61 × 10^−20^
SP-MCTS-CRIPPA-150	**12**	15.5	21.48	100	2.17	3.49	0.94	2.82 × 10^−22^
ACO-15-15	15	17.66	10.46	99.52	1.19	**0.48**	1	3.39 × 10^−12^
ACO-30-30	14	16.46	61.57	99.52	**0.98**	0.69	1	9.16 × 10^−8^
PPACO-30-30	13	***15.22***	60.84	99.52	1.86	2.24	1	–
SL-MCTS-30	13	16.20	**5.34**	**19.90**	3.12	1.17	1	**2.08 × 10^−2^**
**Task 3**								
UCB1-50	42	59.27	29.46	100	10.62	6.02	0.98	2.36 × 10^−47^
UCB1-150	26	48.08	94.42	100	9.74	23.47	1	2.65 × 10^−39^
MCTS-50	33	56.85	28.89	100	13.03	6.76	0.96	8.61 × 10^−38^
MCTS-150	26	47.24	65.84	100	9.18	12.71	1	2.26 × 10^−40^
SP-MCTS-50	42	73.16	42.66	100	19.97	17.76	1	3.66 × 10^−44^
SP-MCTS-150	42	73.16	42.66	100	19.97	17.76	1	1.30 × 10^−35^
SP-MCTS-CRIPPA-50	–	–	–	–	–	–	–	–
SP-MCTS-CRIPPA-150	84	84	172.21	100	–	–	0.04	–
ACO-15-15	17	20.70	**16.62**	90.05	1.75	**1.08**	1	1.65 × 10^−14^
ACO-30-30	**16**	18.9	82.97	98.52	**1.45**	3.09	1	5.67 × 10^−5^
PPACO-30-30	**16**	***17.68***	69.46	99.60	1.55	2.19	1	–
SL-MCTS-30	18	42	24.48	**28.90**	21.89	14.13	0.92	2.82 × 10^−14^

The best value for each evaluation metric is marked in bold.

Table 1 shows that, for the traditional MCTS algorithms, in Task 1, UCB1-50 have the shortest optimal path of 10, with the shortest average path length of 14.1. In Task 2, SP-MCTS-CRIPPA-150 obtained an optimal path length of 12 and a shortest average path length of 15.5, but its success rate in solving problems is 0.94. In Task 3, UCB1-150 has an optimal path of 26 than other traditional MCTS algorithms and an average path length of 48.08. It's worth mentioning that the time consumption of the traditional MCTS algorithm increases significantly as the iteration times increase. For collective intelligence algorithms, ACO-30-30 has the smallest optimal solution and average path length for Task1, at 10 and 10.68 respectively. For Task 2 and Task 3, PPACO-30-30 has the shortest average path length out of all ACO algorithms, which is 15.22 and 17.68 respectively. Compared to traditional MCTS algorithms and collective intelligence algorithms, SL-MCTS-30 only explored 14.69% of the environment in Task 1 and it takes an average of 2.99 s. The quality of SL-MCTS-30's path is only inferior to ACO-30-30 and ACO-15-15. Its time consumption is the least. Its optimal path length is 10, with an average path length of 11.08. Furthermore, the standard deviation of SL-MCTS-30 in terms of path length and time consumption is the lowest among other compared algorithms, at 1.59 and 0.04 respectively. This suggests that the performance of SL-MCTS-30 is more stable. Mann–Whitney *U*-tests were performed to obtain the results between the algorithm with the best Average length (shown in bold italics) and other algorithms. In Task 1, ACO-30-30 is determined to be the best method. The results of the significance test show that there is no significant difference between AS-30-30 and SL-MCTS-30. In Task 2, the optimal solution of SL-MCTS-30 is 13, which is the same as PPACO-30-30, second only to SP-MCTS and SP-MCTS-CRIPPA-150. The average path length of SL-MCTS is 16.20, only 0.7 longer than that of SP-MCTS-CRIPPA-150 and PPACO-30-30, but the exploration space of SL-MCTS is only 19.9%, one fifth of other algorithms. Additionally, the average consumption time for SL-MCTS was the shortest amongst all algorithms, taking only 5.34*s*. PPACO-30-30 is determined to be the best method. The results of the significance test show that there is no significant difference between PPACO-30-30 and SL-MCTS-30. In task 3, the optimal solution of SL-MCTS-30 is 18, which is only second to the ant colony algorithms. Moreover, SL-MCTS explores only 28.90% of the environment space and solves the problem in just 25.48 s, making it a highly efficient algorithm. In conclusion, SL-MCTS with a simulation count of 30 performed significantly better than traditional MCTS and SP-MCTS algorithms with simulation counts of 50 or 150. Its performance is comparable to that of ACO, which is proficient at solving path planning tasks. The experimental results show that under the guidance of the PV-Network, SL-MCTS converges faster than other MCTS algorithms. However, SL-MCTS is considerably more efficient than ACO in terms of time consumption and search space for most tasks, with time consumption of less than half and search space only one fifth that of ACO. It is meaningful to mention that some MCTS algorithms are unable to solve the complex path planning problem (such as SP-MCTS-CRIPPA), mainly because most traditional MCTS algorithms are designed for game scenarios and not proficient at solving path planning tasks. However, with the proposed method in this paper, SL-MCTS has made significant improvements over MCTS algorithms

**Figure 11:**
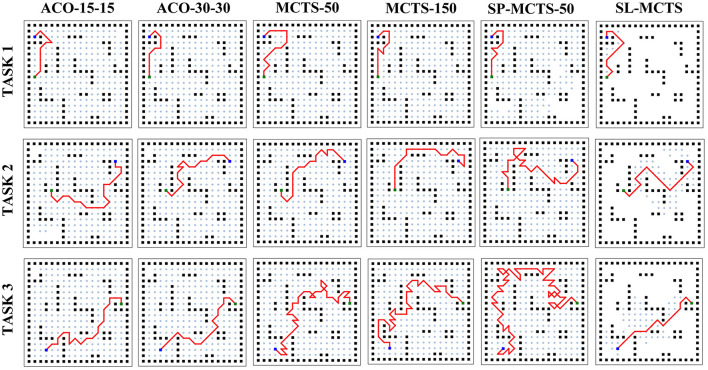
Visualization of path planning results for ACO, MCTS, and SL-MCTS. Black nodes indicate obstacles. Blue nodes indicate origins, green nodes indicate destinations and red lines indicate the found paths. The range visited by the algorithm is marked with light blue nodes.

Figure 11 visualizes the planning results of SL-MCTS, MCTS-50, MCTS-150, SP-MCTS-50, ACO-15-15, and ACO-30-30. Black nodes indicate obstacles, blue nodes indicate origin, green nodes indicate destinations and red lines indicate found paths. The range visited by the algorithm is marked with light blue nodes. The visualization of these algorithms' path planning results presents that the paths of SL-MCTS have fewer inflection points than other traditional MCTS algorithms, and the number of visited nodes is much less than others. This also indicates that the search of SL-MCTS is efficient, and the path of SL-MCTS is reasonable and competitive with other baselines.

#### 3.3.3. Generalization of SL-MCTS

This section aims to evaluate the potential of SL-MCTS in tackling tasks in previously unseen environmental maps. Two sets of experimental maps, each with three different obstacle densities, were constructed based on the two map sizes. Fifty tasks of random starting and ending points were selected on each map to form the test set of each map. The map size is 6 × 6, defined as MAP 1, and 16 × 16, defined as MAP 2. The number of SL-MCTS's simulations is 30. Maps are named Sparse Map 1, Moderate Map 1, Dense Map 1, Sparse Map 2, Moderate Map 2 and Dense Map 2 according to the density of obstacles in the maps (5%, 25%, and 55%). In Table 2, we presented the performance of SL-MCTS by analyzing the ratio of SL-MCTS to MCTS-50 in terms of path length (*Ratio*_*length*_) and time consumption (*Ratio*_*time*_), which were calculated by:


(15) $$\begin{array}{l} Ratio_{length}=\frac{1}{50}\overset{50}{\underset{i=1}{\sum }}\frac{path_{Ai}}{path_{Bi}} \end{array}$$

(16) $$\begin{array}{l} Ratio_{time}=\frac{1}{50}\overset{50}{\underset{i=1}{\sum }}\frac{time_{Ai}}{time_{Bi}} \end{array}$$

where *i* is the number of the testing tasks. *A* represents SL-MCTS and *B* represents MCTS-50. A lower ratio indicates that SL-MCTS performs better than MCTS-50 in terms of path quality or time consumption. The success rate is defined as the proportion of successfully completed tasks to the total number of testing tasks.

**Table 2:** Comparative analysis of path lengths and time consumption for SL-MCTS and MCTS-50 in the test map sets.

	***Ratio*_*length*_**	***Ratio*_*time*_**	**Success rate(%)**
Sparse-Map 1	0.92	0.59	100
Moderate-Map 1	0.94	0.67	100
Dense-Map 1	0.91	0.55	100
Sparse-Map 2	0.84	0.54	100
Moderate-Map 2	0.82	0.58	100
Dense-Map 2	0.64	0.42	100

In Table 2, the success rates of SL-MCTS in maps are 100%. It means that SL-MCTS can successfully tackle the tasks in these unseen environmental maps. The *Ratio*_*length*_ value is about 0.90 on the set of maps for MAP 1 and about 0.76 on that for MAP 2. The *Ratio*_*time*_ value is about 0.5 both on maps 1 and 2. These experiments indicate that SL-MCTS performs significantly better than MCTS-50 in terms of path quality, particularly in environments with a map size of 16. Furthermore, SL-MCTS completes the same task using only half of the time computation required by MCTS-50. SL-MCTS performs better on MAP2 than on MAP1, which may be due to the larger search space and greater number and variety of obstacles on MAP2, making tasks more challenging and enabling SL-MCTS to demonstrate its superior capabilities. In general, these experiments demonstrated that SL-MCTS not only is able to find the tasks' solutions on the new maps but also completes them with half the time required by MCTS-50, particularly for tasks with shorter lengths.

We conducted additional experiments on random maps with different obstacle distributions. By comparing the proposed algorithm's performance in solving the same task in these diverse environmental maps, we further assessed SL-MCTS's ability to adapt to novel environmental maps. We chose two test tasks: one on a map with a size of 6, with a starting point at (0, 0) and an ending point at (5, 5); the other on a map of size 16 with a starting point at (3, 8) and an ending point at (14, 14). The considerable span of both tasks on their respective maps allowed us to examine different obstacle distributions. Tables 3, 4 display the results performed by SL-MCTS on different-sized maps, and these results are compared with those of MCTS-50. The “prior map” in these tables refers to the environmental map utilized for SL-MCTS learning, while the “random map” denotes an environment with a different obstacle distribution compared with “prior map,” which SL-MCTS has unseen before. We have provided more information about the environmental map in the public code repository (Liu, 2023). The test tasks were repeated 50 times per map. This section analyzed the ability of SL-MCTS to handle tasks in new environments by comparing its best and average path lengths, the standard deviation of path lengths (SD-L), average time consumption (average time) and standard deviation (SD-T) of time consumption, and success rate with those of MCTS-50. We also employed the Mann-Whitney U test as a significance test to determine the mean difference between the experimental results for SL-MCTS and MCTS-50 (the best Average length, shown in bold italics).

**Table 3:** Results of SL-MCTS and MCTS on different 6 × 6 maps.

	**Best**	**Average length**	**SD-L**	**Average time**	**SD-T**	**Success rate(%)**	***p*-value**
**Prior map**							
SL-MCTS	6	***8.42***	2.43	0.51	0.35	0.95	–
MCTS-50	9	15.16	4.16	0.86	0.22	1	3.01 × 10^−11^
**Random map1**							
SL-MCTS	7	***9.55***	2.45	0.56	0.34	0.78	–
MCTS-50	16	26.60	12.85	0.78	0.22	1	0.017
**Random map2**							
SL-MCTS	7	***10.45***	2.36	0.68	0.144	0.83	–
MCTS-50	8	13.8	3.42	0.94	0.27	1	1.69 × 10^−10^

The best value is highlighted in italic bold.

**Table 4:** Results of SL-MCTS and MCTS on different 16 × 16 maps.

	**Best**	**Average length**	**SD-L**	**Average time**	**SD-T**	**Success rate(%)**	***p*-value**
**Prior map**							
SL-MCTS	12	***20.16***	13.61	7.12	5.19	0.74	–
MCTS-50	20	33.84	9.09	15.17	4.03	1	5.79 × 10^−8^
**Random map**							
SL-MCTS	15	32.5	15.63	5.11	7.06	0.68	**0.07**
MCTS-50	15	***32.20***	15.05	12.72	6.04	1	—

The best value is highlighted in italic bold.

According to the results in Table 3, SL-MCTS outperforms MCTS-50 in both the “prior map” and new “random map” environments. Specifically, SL-MCTS had a much shorter average path length than MCTS-50, along with a smaller standard deviation in path lengths. This indicates a higher solution quality and lower fluctuation compared to MCTS-50. In addition, SL-MCTS also consumed significantly less time on average than MCTS-50. Furthermore, the results of the significance test in both “random map1” and “random map2” show that there is a significant difference between SL-MCTS and MCTS-50, with SL-MCTS being the best method. Table 4 shows that SL-MCTS's average path length and SD-L in “random map” environments were similar to those of MCTS-50. SL-MCTS's success rate on “random maps” was 0.68 lower than that on the “prior map.” This could be attributed to the excessive density of obstacle distribution between the start and end points, including an obstacle corridor that blocks access between the beginning and the destination. This significantly increases the difficulty of the testing task on the “random map” compared to that on the prior map. The results of the significance test in the “random map” show that there is no significant difference between SL-MCTS and MCTS-50. The results show that SL-MCTS can solve the tasks on the new maps, indicating that the problem-solving ability of SL-MCTS has generalization in unseen environmental maps.

#### 3.3.4. Ablation experiments

**Figure 12:**
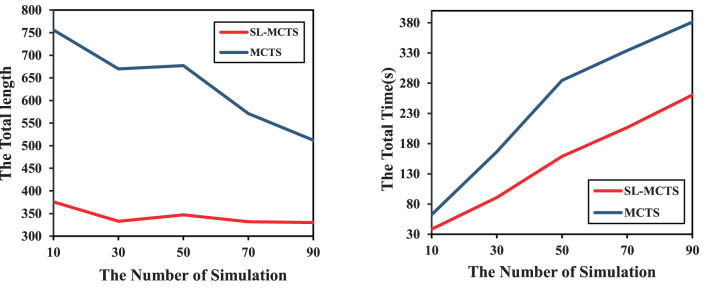
Comparison of the variations in total path length and total time consumption of SL-MCTS and MCTS with different numbers of simulations.

This section presents the effect of PV-Network on the SL-MCTS algorithm with a different number of simulations. Thirty tasks are randomly selected from the 16 × 16 map as a test set. The variation of the total length and the total time consumption of SL-MCTS-30 and MCTS-30 was compared on the test set. As shown in Figure 12, five different simulations (10, 30, 50, 70, and 90) are chosen. The total path lengths of SL-MCTS and MCTS decrease as the number of simulations increases, which means that increasing the number of simulations can improve the quality of MCTS's solution. However, for different simulation numbers, SL-MCTS has significantly shorter path lengths than MCTS, being almost half of MCTS's lengths. Although the time consumption of both algorithms increases with the number of simulation, traditional MCTS algorithms become more time-consuming with higher simulation numbers. And the time consumption of SL-MCTS is consistently lower than MCTS, about two-fifths of MCTS's total time. Experiments show that PV-Network can provide accurate guidance for the search process of SL-MCTS, and SL-MCTS is more efficient in finding higher quality solutions than the traditional MCTS algorithms.

#### 3.3.5. Test on dynamic environmental map

**Figure 13:**
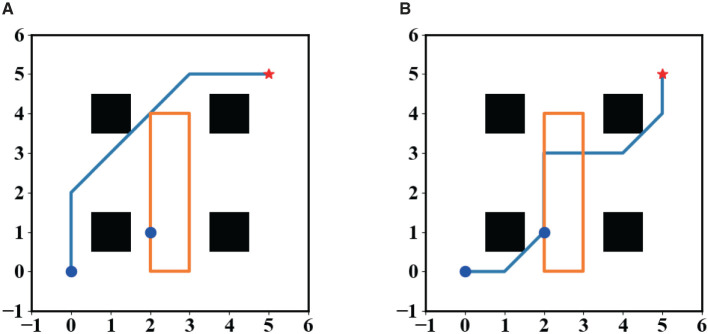
**(A, B)** The path planning results of the robot in a dynamic environment.

Finally, we tested the performance of SL-MCTS in a dynamic obstacle environment to deal with stochastic environments. In addition to the eight actions shown in Figure 1B, the robot's actions included the “wait” action. As shown in Figure 13, there was a dynamic obstacle in the environmental map, which is clockwise, and its movement trajectory was shown as an orange line. The trajectory has the starting point of (1, 2) and four turning points at (4, 2), (4, 4), (0, 3), and (0, 2). The robot's initial position was (0, 0) and the endpoint was (5, 5). Figure 13 shows two trajectories of the robot to deal with this dynamic obstacle. Figure 13A shows the robot successfully reached the destination without colliding with the dynamic obstacle. The robot chooses to bypass the area of the dynamic obstacle to reach the endpoint. Figure 13B shows the trajectory of the robot colliding with the dynamic obstacle at position (3, 3). To avoid collision and task failure, the robot waits in position (3, 2). These experiments demonstrated that SL-MCTS can handle dynamic environments. More related animations have been uploaded to the public repository (Liu, 2023).

## 4. Conclusion

Inspired by the idea of “self-player” for two-player zero-sum games, this paper proposes a self-learning single-player MCTS, named SL-MCTS, to continually enhance the problem-solving ability of agents in single-player scenarios. The main contributions of this paper include constructing the self-learning framework for single-player scenarios and designing an efficient evaluation method to assess the quality of the agent's strategies in the learning process. In the experiment section of this paper, a widely-renowned robot path planning scenario was utilized to validate the efficacy of SL-MCTS. In the self-learning process, the increasing Elo ratings of SL-MCTS show that the “self-learning” method for the single-player task is effective. The performance of SL-MCTS is also compared with that of MCTS, SP-MCTS, SP-MCTS-CRIPPA, and the currently popular collective intelligence algorithms in many different tasks. The results demonstrate that SL-MCTS can find better solutions with fewer iterations than other iteration-based algorithms, which indicates the convergence speed of SL-MCTS is faster. Additionally, in terms of time consumption, the speed of SL-MCTS in solving problems is faster than other comparative algorithms. It can solve problems in less than one-third of the time required by other algorithms. These indicate that the guidance of the PV-Network greatly improves the search efficiency and the resulting quality of SL-MCTS in path planning tasks. Furthermore, we validated the adaptability of SL-MCTS in many new environmental maps. The results show that SL-MCTS can find solutions with better quality in half the time required by MCTS-50. This experiment demonstrates that the problem-solving ability of SL-MCTS is universal across different environmental maps. Finally, we validated SL-MCTS's adaptability in a dynamic environment. The experimental results show that it can successfully solve tasks in dynamically complex scenes. In conclusion, this paper demonstrates that the mechanism of “self-learning” can be applied in single-player scenarios. It provides a new way for the agent with learning capabilities to break through its ceiling of problem-solving ability. Comparative experiments have confirmed that SL-MCTS can alleviate the common issues of slow convergence, poor search quality and inefficient search in traditional MCTS algorithms, while also significantly improving search speed.

In the future, we will further explore applying self-learning with other collective intelligence algorithms. We will also try to extend self-learning to improve the performance of the robotic arms in the continuous action space of the path planning problem.

## Data availability statement

The original contributions presented in the study are included in the article/supplementary material, further inquiries can be directed to the corresponding authors.

## Author contributions

WL provided the original motivation and idea. YL further developed and implemented the idea, conducted the experiments, and produced the initial manuscript. WL and YL engaged in a thorough discussion and revision of this manuscript. YM, KX, and JQ checked the results and provided writing advice for the manuscript. ZG was responsible for the resources and revision of the manuscript and provided financial support. All authors contributed to the article and approved the submitted version.
